# Microvascular Changes in Parkinson’s Disease- Focus on the Neurovascular Unit

**DOI:** 10.3389/fnagi.2022.853372

**Published:** 2022-03-10

**Authors:** Gesine Paul, Osama F. Elabi

**Affiliations:** ^1^Translational Neurology Group, Department of Clinical Sciences, Lund University, Lund, Sweden; ^2^Department of Neurology, Scania University Hospital, Lund, Sweden; ^3^Wallenberg Centre for Molecular Medicine, Lund University, Lund, Sweden

**Keywords:** Parkinson’s disease, vasculature, pericytes, angiogenesis, blood-brain barrier, microglia

## Abstract

Vascular alterations emerge as a common denominator for several neurodegenerative diseases. In Parkinson’s disease (PD), a number of observations have been made suggesting that the occurrence of vascular pathology is an important pathophysiological aspect of the disease. Specifically, pathological activation of pericytes, blood-brain barrier (BBB) disruption, pathological angiogenesis and vascular regression have been reported. This review summarizes the current evidence for the different vascular alterations in patients with PD and in animal models of PD. We suggest a possible sequence of vascular pathology in PD ranging from early pericyte activation and BBB leakage to an attempt for compensatory angiogenesis and finally vascular rarefication. We highlight different pathogenetic mechanisms that play a role in these vascular alterations including perivascular inflammation and concomitant metabolic disease. Awareness of the contribution of vascular events to the pathogenesis of PD may allow the identification of targets to modulate those mechanisms. In particular the BBB has for decades only been viewed as an obstacle for drug delivery, however, preservation of its integrity and/or modulation of the signaling at this interface between the blood and the brain may prove to be a new avenue to take in order to develop disease-modifying strategies for neurodegenerative disorders.

## Brain Vasculature

The brain is a highly oxygen consuming organ and, as a result, has developed a dense network of almost 650 km of microvessels (Pandey et al., 2016). The smallest entity is formed by capillaries that are in close contact with the surrounding parenchyma and allow the gas exchange. This close connection between blood and brain is termed the neurovascular unit (NVU). The NVU consists of endothelial cells, pericytes and the basal lamina forming the microcapillary wall, and cells in the immediate surrounding brain parenchyma including perivascular astrocytes, perivascular microglia and neurons.

### Blood Vessels as Adaptors of Blood Flow

Capillaries of the brain are non-fenestrated vessels regulating the influx of nutrients and oxygen according to the changing demands of the brain (Iadecola, 2017).

The adaption to the brains requirements occurs by neurovascular coupling, matching the local blood supply to the neuronal demand by adjustment of the vascular intraluminal diameter (Carmignoto and Gomez-Gonzalo, 2010). Preservation of the highly balanced homeostasis of the brain’s microenvironment, however, is guaranteed by the BBB.

### Blood Vessels as Gate Keepers at the Blood-Brain Barrier

The BBB is formed by endothelial cells that require close contact with pericytes in order to form tight junctions, by the basal lamina and by astrocytic endfeet (Zhao et al., 2015). The integrity of the BBB is absolutely vital for normal neuronal function. A leaky BBB enables the uncontrolled entry of pathogens, toxins and inflammatory cells into the brain and leads to inflammatory and immune responses. BBB leakage, whether subtle or severe, ultimately leads to neuronal injury, neurodegeneration and accelerates disease progression (Bell et al., 2010; Sweeney et al., 2018a).

### Blood Vessels as Communicators of Signals

As brain capillaries form the contact surface between the blood and the brain, cells at this interface are also the first sensors of systemic changes such as metabolic imbalances, systemic inflammation, circulating pathogens, changes in oxygen tension etc. In particular brain pericytes have been identified as first responders to systemic inflammation mediating signals from the blood onto the neighboring parenchyma cells (Duan et al., 2018). Vascular pathology and changes in cell signaling at and across the BBB may be the interface linking systemic risk factors (e.g., diabetes or chronic inflammation) to neuroinflammation and neurodegeneration (see section “Metabolic Disorders and Vascular Changes in Parkinson’s Disease”).

## Vascular Changes in Parkinson’s Disease

Blood vessel alterations, BBB disruption and cerebral blood flow abnormalities are a common denominator of several neurodegenerative disorders and have been described in Alzheimer’s disease (Sweeney et al., 2018b), amyotrophic lateral sclerosis (Zhong et al., 2008; Garbuzova-Davis and Sanberg, 2014; Winkler et al., 2014), Huntington’s disease (Padel et al., 2018) and Parkinson’s disease (PD). There is a growing appreciation that vascular alterations can contribute to disease onset and aggravate the neurodegenerative process as some vascular changes already occur before the onset of neuronal loss or behavioral deficits in animal models of the respective disease (Sagare et al., 2013; Winkler et al., 2014; Padel et al., 2016; Elabi O. et al., 2021).

Here we particularly outline the different histological vascular changes reported in patients with PD and summarize the evidence for vascular alterations from animal models of PD. This minireview does not cover the role of hypoperfusion or white matter lesions in the pathogenesis of PD.

### Parkinson’s Disease

Parkinson’s disease (PD) is the second most common neurodegenerative disorder and one of the fastest growing neurological diseases. In 2015, PD affected 6.9 Million people worldwide, a number expected to double by 2040 due to the aging population (Dorsey and Bloem, 2018).

The progressive degeneration of the nigrostriatal system gives rise to the typical clinical symptoms rigidity, bradykinesia and resting tremor (Fearnley and Lees, 1991). In PD, dopaminergic neurons in the substantia nigra pars compacta (SNpc) are degenerating and the histopathological hallmark is the formation of Lewy bodies containing aggregated alpha-synuclein (α-syn) (Spillantini et al., 1997). Although PD is associated with these distinct histological changes, concomitant pathological alterations might sustain or aggravate the neuronal degeneration. This is particularly relevant as there is currently no therapy available that intervenes with the ongoing disease process. In this context, any contributor to the disease is important to elicit.

### The Microvascular Environment in Parkinson’s Disease

**FIGURE 1:**
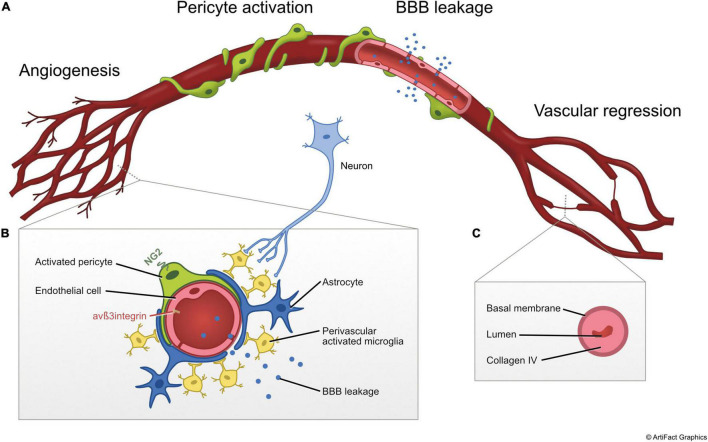
Illustration of vascular changes observed in post mortem tissue and preclinical models of PD. **(A)** Simplified diagram illustrating the main vascular alterations in non-chronological order including (1) angiogenesis, (2) pericyte activation, (3) BBB leakage, and (4) vascular regression. **(B)** Cross-sectional diagram of the NVU during the angiogenic response. **(C)** Cross-sectional diagram of string vessel showing the collapsed basement membrane labeled with collagen-IV and absence of endothelial cells during vascular regression stage. The process of vascular regression includes loss of pericytes and signaling between pericytes and endothelial cells, causing destabilization of endothelial cells and endothelial cell death. NG2, neuron-glial antigen 2.

Almost 90 years ago, it was described that the capillary network in the SNpc is considerably denser than in the adjacent SN zona reticulata (Finley, 1936). Under normal conditions, a distinct tight pattern of neuron-capillary associations is observed in the SNpc. However, in PD, these close contacts between dopaminergic neurons and microvessels are lost leaving an “empty space” (Issidorides, 1971). Based on these early observations it was postulated that modifications of the vascular microenvironment of dopaminergic neurons may alter the availability of nutrients or lead to accumulation of toxic compounds in the immediate vicinity of these cells. Later, vascular alterations in PD were described more in detail ranging from signs of angiogenesis to BBB leakage and vascular regression (Figure 1).

### Evidence for Angiogenesis in Parkinson’s Disease

Angiogenesis refers to the formation of new blood vessels in adulthood. In the adult brain it usually occurs in response to hypoxia or inflammation (Tahergorabi and Khazaei, 2012). Angiogenesis can be recognized by an increase in vascular density and branching points, proliferation of vascular cells, expression of angiogenic markers or an increase in angiogenic molecules in the brain or cerebrospinal fluid (CSF).

#### Angiogenic Microvessels in Parkinson’s Disease

First evidence for pathological angiogenesis in PD comes from studies in the 90’s observing a 2.5-fold increase in the number of endothelial cells in PD brains (Faucheux et al., 1999) and an increased number of integrin αvβ3-positive vessels, an adhesion molecule that is present on angiogenic vessels (Brooks, 1996) in the SNpc, the locus coeruleus and the putamen, all regions which are affected in PD (Desai Bradaric et al., 2012).

Similar findings reporting angiogenesis in several brain regions were made in the 6-hydroxydopamine (6-OHDA) PD model in rats (Carvey et al., 2005) and mice (Elabi O. F. et al., 2021), in an experimental model of L-DOPA-induced dyskinesias (Westin et al., 2006) and in the 1-methyl-4-phenyl-1,2,3,6-tetrahydropyridine (MPTP) monkey model (Barcia et al., 2005).

Even though toxin-induced models are useful to study several aspects of PD, they do not reflect the slowly progressive nature of PD pathology. Using a human α-syn overexpression mouse model that recapitulates the progressive aggregation of human α-syn (Hansen et al., 2013), we confirmed an increase in vessel density indicating angiogenesis at the moderate stage of the animal model. In late-stage animals, however, the vessel density was significantly reduced suggesting dynamic and stage-dependent vascular changes in PD (Elabi O. et al., 2021).

#### Pathological Pericyte Activation

Interestingly, at the early stage of α-syn-aggregation, we observed an activation of pericytes that was preceding changes in vessel density and behavioral deficits (Elabi O. et al., 2021). Pericytes line the entire microvasculature of the brain and have an important function in angiogenesis (Stapor et al., 2014). Activation of pericytes leads to changes in morphology and marker expression. Capillary pericytes generally have a flat cell soma with extensive longitudinal and thin processes (Dore-Duffy and Cleary, 2011). However, under pathological conditions, pericytes acquire a more bulging cell soma with shorter processes, typical of activated and migratory pericytes (Dore-Duffy and Cleary, 2011; Ozen et al., 2014). This pattern is predominantly seen following injury and during the early stages of angiogenesis and often associated with expression of markers such as NG2 and/or RGS5 (Ozerdem and Stallcup, 2004; Berger et al., 2005). Angiogenesis requires first pericyte detachment from the vessel wall, allowing endothelial sprouting and then subsequent pericyte recruitment for stabilization and maturation of the vasculature (Kamouchi et al., 2012). We have previously shown that pericytes are activated in the 6-OHDA PD model (Padel et al., 2016), and that pathological pericyte activation is a feature of also other neurodegenerative disorders (Padel et al., 2018).

Pericytes are one of the first responders to brain hypoxia (Gonul et al., 2002; Duz et al., 2007) and to systemic inflammation (Duan et al., 2018). Importantly, pericytes alter their signaling toward a pro-inflammatory secretome when activated (Rustenhoven et al., 2017; Gaceb and Paul, 2018; Gaceb et al., 2018). Interestingly, a direct observation that α-syn can activate pericytes comes from an *in vitro* study where exposure to monomeric α-syn leads to secretion of high amounts of pro-inflammatory molecules in pericytes that in turn mediated hyperpermeability in endothelial cells resulting in BBB leakage (Dohgu et al., 2019).

Thus, it is conceivable that pericyte activation may form the starting point of vascular alterations and a cascade of pathological signaling events in the NVU in PD.

#### Angiogenic Molecules

Findings indicating angiogenesis in PD are supported by reports showing an upregulation of the pro-angiogenic molecule Vascular Endothelial Growth Factor (VEGF) in the SNpc of PD patients (Wada et al., 2006; Yasuda et al., 2007; Lan et al., 2021) and non-human primates (Barcia et al., 2005). Increased levels of soluble VEGF receptor-2 and placental growth factor, and lower levels of angiopoietin 2 (an anti-angiogenic molecule) were detected in the CSF of PD patients (Janelidze et al., 2015). In this study, angiogenesis markers in the CSF were associated with microbleeds and white matter lesions on imaging, suggesting abnormal angiogenesis in PD (Janelidze et al., 2015). Further strengthening these findings, a recent study demonstrated CSF changes in miRNAs regulating pathways of angiogenesis and BBB components in PD patients with moderate disease, implying impairment of these pathways as part of the progression of PD (Fowler et al., 2021).

### Blood-Brain Barrier Dysfunction in Parkinson’s Disease

#### Blood-Brain Barrier Dysfunction in Parkinson’s Disease Animal Models

Angiogenesis is a double-edged sword as newly formed vessels are immature and can lead to BBB leakage, especially when pericyte recruitment is impaired.

Indeed, a dysfunctional BBB has been demonstrated in a number of different PD models showing leakage of albumin and other tracers into the brain parenchyma (Carvey et al., 2005, 2009; Westin et al., 2006; Zhao et al., 2007; Chen et al., 2008), increased entry of drugs (Carta et al., 2006; Westin et al., 2006) and infiltration of peripheral immune cells otherwise are prevented from crossing the BBB (Benner et al., 2008; Brochard et al., 2009; Reynolds et al., 2010). Few studies have not been able to confirm BBB leakage, likely due to the methods used (Astradsson et al., 2009; Elabi O. F. et al., 2021).

A study using the A53T PD mouse model showed that the expression of tight junction-related proteins at the BBB decreased leading to increased vascular permeability (Lan et al., 2021). When we examined the temporal dynamics of BBB leakage in another progressive α-syn-PD mouse model (Elabi O. et al., 2021), we detected significant extravascular fibrinogen accumulation already at the early stage preceding behavioral deficits (Elabi O. et al., 2021).

#### Blood-Brain Barrier Dysfunction in Parkinson’s Disease Post Mortem Tissue

The evidence of an impaired BBB in PD from animal models is validated by compelling post mortem studies in PD using a variety of different methods. Gray and Woulfe (2015) found a sevenfold increase in extravasated erythrocytes, a threefold increase in hemosiderin depositions (often grouped around capillaries), a significant perivascular deposition of hemoglobin (8.6-fold increase) and a 9.4-fold increase in extrasudated fibrinogen in the striatum of PD patients compared to controls. Greater fibrinogen accumulation (Yang et al., 2015), higher IgG leakage and loss of tight junction proteins (Pienaar et al., 2015) were also reported in other autopsy studies.

Similarly, a 10-fold increase of extravascular CD4^+^ and CD8^+^ lymphocytes has been shown particularly in the SNpc in post mortem PD brain tissue (Brochard et al., 2009) demonstrating pathological immune cell infiltration across the BBB.

#### Blood-Brain Barrier Dysfunction Examined in the Cerebrospinal Fluid

In line with post mortem findings, CSF studies have shown BBB leakage as indicated by increased levels of CSF albumin in PD correlating with the severity of the disease (Pisani et al., 2012), or with the level of angiogenic factors in the CSF (Janelidze et al., 2015).

#### Blood-Brain Barrier Dysfunction Using *in vivo*-Imaging

Blood-brain barrier dysfunction in PD patients *in vivo* is more difficult to study. Using positron-emission tomography (PET), progressive BBB impairment has been shown in the midbrain of PD patients as seen by an increased uptake of the tracer ^11^C-verapamil indicating impairment of the BBB efflux pump P-glycoprotein (Kortekaas et al., 2005; Bartels et al., 2008) and analysis of dynamic contrast-enhanced magnetic resonance images revealed higher BBB leakage in PD patients (Al-Bachari et al., 2020), whereas a study using rubidium-82-PET could not detect BBB leakage in PD patients (Fujita et al., 2021).

### Vascular Regression

Angiogenesis and BBB leakage are not the only vascular pathology that has been observed in PD. Signs indicating vascular regression come from reports showing endothelial degeneration, decrease in vessel length and number of branching points and increase in vessel diameter in the SN of PD patients compared to age-matched controls (Guan et al., 2013; Yang et al., 2015). In addition, PD patients had higher numbers, density and total length of “string vessels” when compared to controls (Yang et al., 2015). String vessels are linked to endothelial cell degeneration leaving empty collapsed basal membrane tubes that do not take part in perfusion (Brown, 2010). Using electron microscopy, Farkas et al. (2000) also demonstrated basal membrane thickening in cerebral capillaries in PD.

Vascular regression likely indicates a later stage of vascular pathology in PD. When studying the temporal dynamics of vascular changes, we noted that early pericyte activation and BBB leakage were followed by angiogenesis, whereas vascular rarefication did not occur until the late stage of the PD model (Elabi O. et al., 2021). Thus, the microvasculature in PD might undergo both, an angiogenic and pruning vascular response, whereby occurrence of BBB leakage could be an early event, followed by the attempt for neovascularization at the moderate stage of the disease and vascular degeneration as a sign of late-stage disease. In the α-syn-PD mouse model we observed colocalization of α-syn and phosphorylated α-syn with endothelial cells at all stages, which suggests a direct involvement of α-syn in the vascular pathological mechanism in addition to a pathological stimulation of pericytes (Elabi O. et al., 2021).

### Inflammation and Vascular Pathology

#### Microglia

In PD, neuroinflammation is a well-known pathology as documented by increased numbers of activated microglia in PD brains (Mcgeer et al., 1988; Croisier et al., 2005; Zhang et al., 2005; Whitton, 2007; McGeer and McGeer, 2008) and increased levels of pro-inflammatory molecules in the CSF of PD (Blum-Degen et al., 1995).

Several studies demonstrating an increased activation of microglia also reported BBB disruption in these PD models (Carvey et al., 2005; Zhao et al., 2007; Elabi O. et al., 2021). The interactions between microglia and blood vessels are complex: Microglia are likely activated by the parenchymal leakage of plasma proteins (Merlini et al., 2019), on the other hand, activated microglia may also induce angiogenesis and vascular leakage *via* the release of inflammatory and pro-angiogenic molecules (Naldini and Carraro, 2005; Ritzel et al., 2015; Haddick et al., 2017; Salter and Stevens, 2017; Chen et al., 2019). The proinflammatory cytokines cause a reduction in the expression of tight junction proteins and increase matrix metalloproteinase-3 and −9, which affect the BBB integrity (Raymond et al., 2016; Bonetti et al., 2019; Edwards et al., 2020).

Recently, we have observed that activated microglia are highly localized particularly in the perivascular space in PD (Elabi O. F. et al., 2021). It has been suggested that perivascular microglia have a dual effect on vessels, maintaining vascular integrity under normal conditions, but phagocytosing the vessel and impairing BBB integrity under prolonged inflammation (Haruwaka et al., 2019). The reason for this increase in activated perivascular microglia in PD is not known, however, we noted the level of perivascular microglia to be strongly associated with the number of pericytes (Elabi O. F. et al., 2021), suggesting a possible interaction of these two cell types at the vascular border.

#### Other Inflammatory Cell Types

Within the NVU, also astrocytes can release pro-inflammatory cytokines and angiogenic molecules that can affect vascular function (Lee et al., 2010; Barcia et al., 2011; Kam et al., 2020). Several studies have highlighted the role of astrocytes in the control of vascular function, *via* e.g., cross-talk with microglia (Wang et al., 2014; Ni et al., 2018).

Similarly, pericytes can produce a variety of inflammatory and angiogenic molecules (Gaceb and Paul, 2018; Gaceb et al., 2018). Activation of pericytes specifically *via* α-syn stimulates release of cytokines and increases expression MMP9 that lead to an increased EC permeability (Dohgu et al., 2019), placing pericytes as mediators between α-syn and BBB disruption.

### Metabolic Disorders and Vascular Changes in Parkinson’s Disease

An increasing number of studies suggest an association between neurodegeneration and metabolic diseases. Epidemiological evidence indicates that diabetes is also a risk factor and a negative prognostic factor for PD (Cereda et al., 2011; Pagano et al., 2018; Heinzel et al., 2019; Sergi et al., 2019; Chohan et al., 2021). The link between metabolic dysfunction and neurodegeneration in PD is further strengthened by studies demonstrating a beneficial effect of anti-diabetic medication in PD and PD models (Foltynie and Athauda, 2020). In particular, Exenatide, a glucagon-like peptide-1 (GLP-1) receptor agonist, has shown neuroprotective effects in preclinical models of PD and entered clinical trials (Athauda et al., 2017).

Even though a number of hypotheses has been put forward to what is leading to this aggravation of PD in the presence of diabetes (Sergi et al., 2019), not much attention has been paid to the fact that diabetes and PD both share pathological microvascular alterations in the brain. Similar to the retinal and renal complications, diabetes is associated with signs of cerebral vascular proliferation and progressive BBB disruption (Starr et al., 2003; Huber et al., 2006; Salameh et al., 2016; Machida et al., 2017; Takechi et al., 2017; Rom et al., 2019; Yamamoto et al., 2019). We have examined the interactions of type 2 diabetes (DMT2) and PD at the microvascular interface and shown that DMT2 in combination with a PD lesion resulted in a significant depletion of pericytes, and reduced interactions between microvessels and perivascular microglia which was associated with a lack of the angiogenic response seen in toxin-induced models (Elabi O. F. et al., 2021). It is conceivable that the diabetic state inhibits the attempt of compensatory angiogenesis seen in PD and accelerates the vessel changes toward a later stage of vascular regression.

## Discussion and Outlook

Current evidence points to a dynamic evolution of multiple vascular changes in PD (Figure 1). These changes might start with pathological pericyte activation and subtle BBB leakage, continue with compensatory angiogenesis that then fails and cumulates in vascular regression. What constitutes the initiator of these events still remains unknown, but their occurrence is certainly contributing to a disturbed neuronal microenvironment. Interventions stabilizing the vasculature and preventing the progression of BBB dysfunction are clearly indicated. Treatment with platelet-derived growth factor (PDGF-BB), a growth factor required for pericyte recruitment and vessel maturation (Jain and Booth, 2003), not only induced neurorestoration and behavioral recovery in PD animal models (Zachrisson et al., 2011; Padel et al., 2016), but also normalized the number of activated pericytes (Padel et al., 2016) and changed the inflammatory secretome of pericytes toward a trophic factor pattern *in vitro* (Gaceb et al., 2018). PDGF-BB has shown safety and tolerability in a phase I/IIa clinical trial in PD patients (Paul et al., 2015; Paul and Sullivan, 2019). It now remains to be seen whether approaches targeting vascular pathology, pericyte activation and vascular signaling at the BBB can modify the progression of the disease.

## Author Contributions

GP and OE wrote the manuscript. Both authors contributed to the article and approved the submitted version.

## Conflict of Interest

The authors declare that the research was conducted in the absence of any commercial or financial relationships that could be construed as a potential conflict of interest.

## Publisher’s Note

All claims expressed in this article are solely those of the authors and do not necessarily represent those of their affiliated organizations, or those of the publisher, the editors and the reviewers. Any product that may be evaluated in this article, or claim that may be made by its manufacturer, is not guaranteed or endorsed by the publisher.
